# Paternal Retrieval Behavior Regulated by Brain Estrogen Synthetase (Aromatase) in Mouse Sires that Engage in Communicative Interactions with Pairmates

**DOI:** 10.3389/fnins.2015.00450

**Published:** 2015-12-15

**Authors:** Shirin Akther, Zhiqi Huang, Mingkun Liang, Jing Zhong, Azam A. K. M. Fakhrul, Teruko Yuhi, Olga Lopatina, Alla B. Salmina, Shigeru Yokoyama, Chiharu Higashida, Takahiro Tsuji, Mie Matsuo, Haruhiro Higashida

**Affiliations:** ^1^Department of Basic Research on Social Recognition, Kanazawa University Research Center for Child Mental DevelopmentKanazawa, Japan; ^2^Department of Biochemistry, Medical Pharmaceutical and Toxicological Chemistry, Krasnoyarsk State Medical UniversityKrasnoyarsk, Russia

**Keywords:** parental behavior, paternal care, brain aromatase, immunoreactivity, communicative interaction, mouse

## Abstract

Parental behaviors involve complex social recognition and memory processes and interactive behavior with children that can greatly facilitate healthy human family life. Fathers play a substantial role in child care in a small but significant number of mammals, including humans. However, the brain mechanism that controls male parental behavior is much less understood than that controlling female parental behavior. Fathers of non-monogamous laboratory ICR mice are an interesting model for examining the factors that influence paternal responsiveness because sires can exhibit maternal-like parental care (retrieval of pups) when separated from their pups along with their pairmates because of olfactory and auditory signals from the dams. Here we tested whether paternal behavior is related to femininity by the aromatization of testosterone. For this purpose, we measured the immunoreactivity of aromatase [cytochrome P450 family 19 (CYP19)], which synthesizes estrogen from androgen, in nine brain regions of the sire. We observed higher levels of aromatase expression in these areas of the sire brain when they engaged in communicative interactions with dams in separate cages. Interestingly, the number of nuclei with aromatase immunoreactivity in sires left together with maternal mates in the home cage after pup-removing was significantly larger than that in sires housed with a whole family. The capacity of sires to retrieve pups was increased following a period of 5 days spent with the pups as a whole family after parturition, whereas the acquisition of this ability was suppressed in sires treated daily with an aromatase inhibitor. The results demonstrate that the dam significantly stimulates aromatase in the male brain and that the presence of the pups has an inhibitory effect on this increase. These results also suggest that brain aromatization regulates the initiation, development, and maintenance of paternal behavior in the ICR male mice.

## Introduction

A stable and well-functioning human family that comprises two parents with a child or children is most secure, wherein the role of the father is highly significant in addition to the essential role of the mother (Waldfogel et al., 2010). However, the physical absence of the father from home due to many reasons is seen as a major problem faced by these families (Amato, 2005; Amato and Anthony, 2014). To understand this more clearly, we require a neuroscientific elucidation of the parent–infant relationship at the behavioral and neuroendocrinological levels (Leuner et al., 2010; Weisman et al., 2013). Although becoming a mother leads to a wide array of remarkable behavioral changes driven by a combination of neuroendocrine and experiential factors, such as oxytocin and steroid hormones, far fewer factors are known to be involved in becoming a father (Onyango et al., 2003; Brunton and Russell, 2010; Higashida et al., 2012; Okabe et al., 2013; Pollet et al., 2013; Rilling, 2013; Dulac et al., 2014; Tsuda et al., 2014).

It is well-known that steroid hormones have a wide variety of effects on reproductive and non-reproductive functions (Lephart, 1997; Pfaff et al., 2000; Choleris et al., 2003). These effects are mediated by slow- and long-lasting genetic actions or rapid and transient non-genomic activities at the physiological and behavioral levels (Vasudevan et al., 2005; Balthazart et al., 2011; Singh et al., 2013). Testosterone secreted from the testis reaches the brain as a prohormone and functions via its conversion into estrogen in nervous tissues (Forlano et al., 2006; Azcoitia et al., 2011). This local conversion into estrogen is catalyzed by brain aromatase [cytochrome P450 family 19 (CYP19); Jenkins et al., 1993; Sun et al., 1998; Crider et al., 2014]. Aromatization appears to be important in modulating social behaviors such as sex, aggression, and paternal behaviors (Forlano et al., 2006; Charlier et al., 2010; Cornil et al., 2013; Yang et al., 2013; Unger et al., 2015). Intriguingly, studies on aromatase knockout mice have demonstrated a critical role of aromatase in parental behavior, wherein knockout males killed their own pups (infanticide by sires ranged from 0 to 70%), attacked females at an increased frequency (three fold more often), and rarely retrieved and nursed pups (20% of that in the wild types; Matsumoto et al., 2003; Honda et al., 2011). Aromatase is located in the hypothalamus and limbic system, including the medial preoptic area (mPOA), bed nucleus of the stria terminalis, ventromedial hypothalamus, medial amygdala (AMY), and many other areas (Beyer et al., 1994; Shinoda et al., 1994; Veney and Rissman, 2000). These studies suggest that brain aromatase may have versatile functions in addition to regulating sex behavior, such as brain sexual dimorphism (Hutchison et al., 1995; Yang et al., 2013) and parental behavior in response to social stimuli (Trainor and Marler, 2002; Cornil et al., 2006; Honda et al., 2011).

Therefore, it would be interesting to determine whether aromatase is one of the critical factors involved in paternal care (parental behavior) and the father–infant relationship (Lonstein et al., 2002). To address this question, we hypothesized that sensitization due to paternity (Rosenblatt, 1967) is associated with rapid estrogen synthesis. Thus, if there is an increase in aromatase, we can investigate the functional role of aromatase in paternity plasticity, particularly with respect to neuronal circuit activation to initiate maternal behavior in non-monogamous sires. Here we quantified the expression of aromatase immunoreactivity in the brains of sires from the ICR mouse because we previously showed that the paternal retrieval behavior in these sires is induced after being separated from pups along with pairmates in separate cages for 10–30 min (Akther et al., 2013, 2014; Fujimoto et al., 2013; Liu et al., 2013; Liang et al., 2014).

## Materials and methods

### Animals

Male and female Slc:ICR mice were obtained from Japan SLC, Inc. (Hamamatsu, Japan) via a local distributor (Sankyo Laboratory Service Corporation, Toyama, Japan). The ICR mice were originally obtained from Charles River Laboratories in 1965 and since then bred in Japan with the alternative name Swiss CD1. The offspring of these mice were born in our laboratory colony, weaned at 21–28 days of age, and housed in same-sex groups of 3–5 animals until pairing (Liu et al., 2013). The animals were paired and kept in our laboratory under standard conditions (24°C; 12-h light/dark cycle, lights on at 08:00) with *ad libitum* food and water. The mice were housed together continuously in standard mouse maternity cages. All of the animal experiments were conducted in accordance with *the Fundamental Guidelines for Proper Conduct of Animal Experiment and Related Activities in Academic Research Institutions* under the jurisdiction of the Ministry of Education, Culture, Sports, Science and Technology of Japan, and they were approved by the Committee on Animal Experimentation of Kanazawa University.

**Figure 1:**
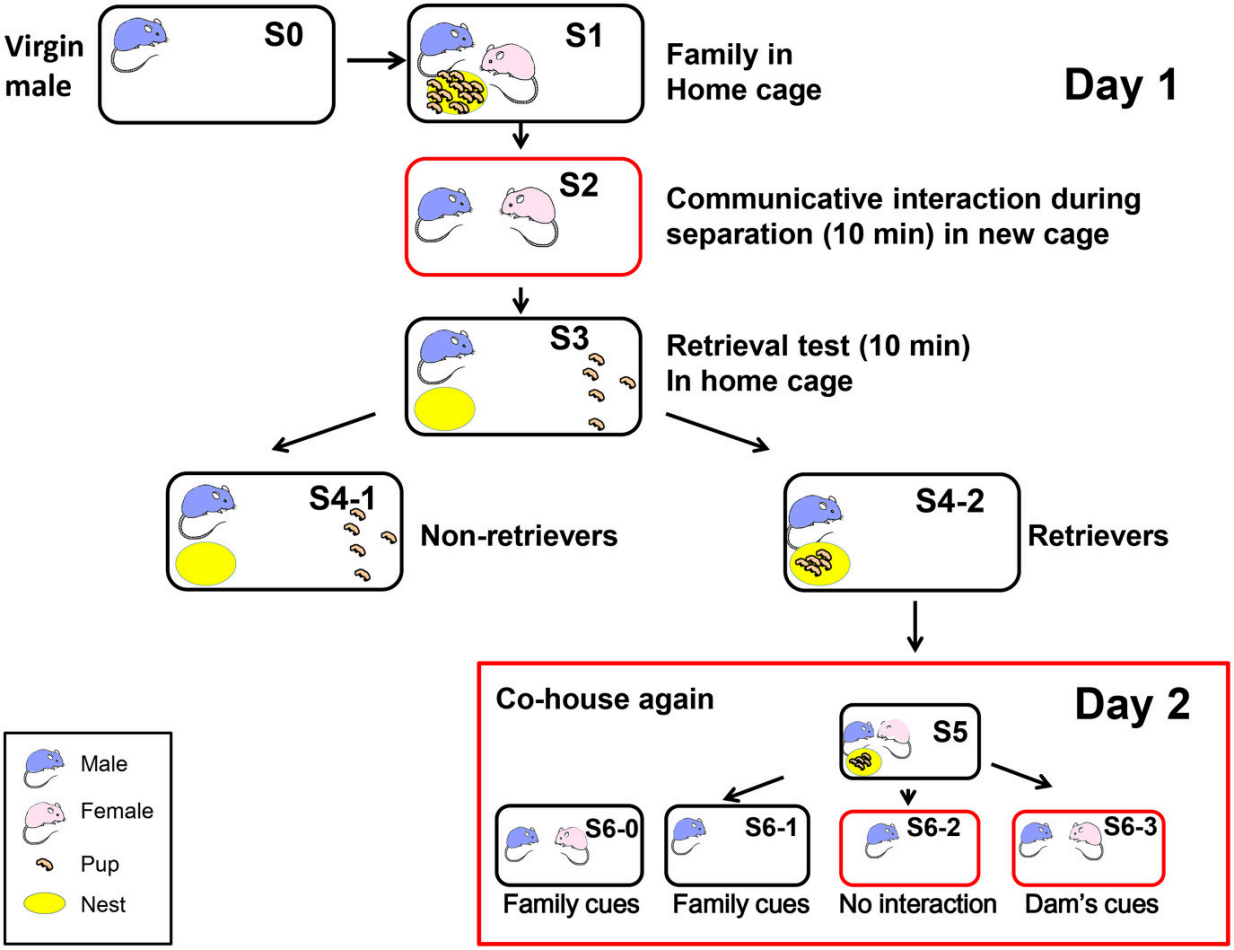
**Schematic showing the different housing conditions and retrieval experiments in the paternal behavior test**. One adult virgin male (S0, blue) and female (pink) were co-housed and maintained until parturition. They were housed together with their biological pups (S1 and S5) in their home cage (black circle). The sire was separated from his pups and pairmate in the home cage (S6-1) or a new cage (red circle; S6-2) for 10 min. The sire was also separated from his pups but placed along with his mate dam in a new cage (S2 and S6-3). Retrieval by the sires during 10 min was examined for five selected pups out of the litters, which were placed in a remote area away from the nest (yellow; S3). The pups in the nest represented retrieval (S4-2) and those outside the nest represented no retrieval of pups to the nest (S4-1) by the sires, retrievers, and non-retrievers. At step S6, the mice were maintained for 30 min before being sacrificed.

Virgin males (S0 in Figure 1) and females were paired for 45–55 days and housed together continuously in a standard mouse maternity cage until after the delivery of their pups (average litter size = 13.5 ± 0.67, *n* = 30; Liang et al., 2014) on postnatal days 3–5. Each of the family units comprised the new sire and dam and their first litter (S1 in Figure 1).

To select pup care-positive sires (retrievers), the parents were separated from the pups and kept in separate cages (environments) for 10 min (S2; Liu et al., 2013). Previously, we detected communicative interactions between the dam and the sire via 38-kHz ultrasonic vocalizations and unidentified pheromones (Liu et al., 2013). Five pups were selected randomly from the litter and placed individually at a site remote from the nest in the original family cage (S3). After isolation for 10 min in the new cage, each sire was returned to its home cage with five biological pups to assess the parental retrieval behavior (S3). The percentage of sires that exhibited retrieval behavior was quantified during 10 min after being reunited with their pups (S4-1 or S4-2; Zhong et al., 2014). The sires that carried all five of their pups to the original nesting place or within two-thirds of the distance between the nest and the remote location, were defined as retrieval-positive sires or retrievers (S4-2; about 60–70% of then sires tested) (Liang et al., 2014). First, we selected paternal behavior-positive sires (retrievers, S4-2 families in Figure 1) to obtain a homogenous sire group, which exhibited pup retrieval during the parental behavior test of the communicative interaction paradigm on the first experimental day (S1–S4).

On the following day (day 2 in Figure 1), we exposed the retrievers to three different conditions and separated them from their pups in the nursing cage (Figure 1 S5): (1) removing the pups (S6-0) or the dam and pups, thus leaving the sire alone in the old cage (S6-1); (2) separating the sire alone in a new cage (S6-2); and isolating the sire and pairmate together in a new cage (S6-3). Based on our previous experiments (Liu et al., 2013; Liang et al., 2014; Zhong et al., 2014), we assumed that the sires left in the old cage (S6-0 and S6-1) could still receive family cues but no ultrasonic vocalizations from the dams (Liu et al., 2013), whereas the sires separated in new cages (S6-2) would have no family cues; however, they would have cues from the mate dam (S6-3) when paired together, in addition to being in an entirely new cage environment. Therefore, we initially compared sires under three conditions (S6-1, S6-2, and S6-3). After 10 and 30 min of co-housing with the mate, the sire was sacrificed immediately and the brain was fixed. The sires that did not satisfy the behavioral definition described by Liu et al. (2013) were designated as retrieval-negative sires or non-retrievers (S4-1). The behavioral tests were conducted at 10:00–15:00 h in a randomly mixed sequence of experimental groups.

Parental males were rendered deaf or anosmic using 0.3–0.5 ml of soft wax (pouring 60°C melted paraffin wax; Histosec DMSO-free, Merck Japan, Tokyo) or via the intranasal infusion of 0.06 ml 5% ZnSO_4_ (Wako Chemical Co., Osaka, Japan) to obtain sensory deprivation conditions, as previously described (Liu et al., 2013). We examined the effectiveness of these procedures as follows. Anosmia was confirmed by the loss of a preference for pure water intake compared with isovaleric acid solution, and histologically by the ablation of the olfactory epithelium. Deafening was tested based on the acoustic startle response. The level of deafness was less than 20% of the control based on the normalized ratio relative to the control (Liu et al., 2013).

### Aromatase immunostaining

Sires were maintained for 30 min in different separation conditions before they were anesthetized and perfused intracardially with cold phosphate-buffered saline (PBS) followed by cold 4% paraformaldehyde (PFA) in PBS. Their brains were removed and fixed in 4% PFA solution overnight at 4°C. Sections were pre-incubated in blocking solution (3% bovine serum albumin and 0.3% Triton X-100 in PBS) for 1 h and then incubated overnight with an antibody against cytochrome p450 (sc-100UG, 1:400; Assay Biotechnology Company Inc., Sunnyvale, CA) in blocking solution. After three washes with washing buffer, the sections were incubated with goat anti-rabbit IgG antibody coupled to Alexa Fluor 488 (Invitrogen, Carlsbad, CA) and DAPI (Wako, Osaka, Japan) in blocking solution for 1 h at room temperature.

Previously, we demonstrated that after interactive communication between sires and dams, the neurons of retrieval pup care-positive sires were excited in the mPOA (0.02 mm from the bregma; Franklin and Paxinos, 2008), ventral tegmental area (VTA, −3.08 mm from the bregma), nucleus accumbens (NAcc, 1.10 mm from the bregma), and ventral pallidum (VP, 0.62 mm from the bregma), which was determined by monitoring c-Fos protein expression (Zhong et al., 2014). In addition to these four regions, we examined the AMY (-1.70 from the bregma), dorsal lateral septum (LSD, 0.62 mm from the bregma), CA3 region of the hippocampus (CA3, −1.70 mm from the bregma), ventral medial hypothalamus (VMH, −1.70 mm from the bregma), and prefrontal cortex (PFC, 2.94 mm from the bregma).

Images for quantitative analysis were obtained using an Olympus IX71 inverted microscope (Tokyo, Japan), which was equipped with a cooled CCD camera (Cool SNAP HQ2; Roper Scientific, Tucson, AZ). We measured the fluorescence intensity in aromatase-immunoreactive cells in each brain section using Metamorph software (Molecular Devices, Sunnyvale, CA). We only counted the aromatase-positive cells within a specific size range and above a constant threshold level of staining as follows: fluorescence diameter < 15 μm and intensity >50 (arbitrary units, a.u.). The total intensity of aromatase fluorescence within the area of imaged section was measured. The same square measures are used between the samples, but different between the regions of interest. Average intensity of aromatase fluorescence in the area was within a 1.2-fold range between the animals that are treated in the same experimental conditions. The fluorescence intensity was used as an indicator of the aromatase enzyme activity because it is known that there is a linear relationship between the aromatase enzyme activity and the immunointensity per cell in human cell lines (Suzuki et al., 1994).

The effect of letrozole was usually examined in the mPOA of sires at 60 min after intraperitoneal injection (1 mg/kg of body weight). The number of nuclei with aromatase-immunoreactivity in brain samples prepared from experimental sires were measured by a confocal laser scanning microscope (Fluoview FV10i, Olympus). We only counted the aromatase-positive cells with fluorescence dots of diameter >5 μm at the constant contrast.

### Statistical analysis

Data were analyzed using One-way or Two-way analysis of variance (ANOVA). *Post-hoc* comparisons were conducted only when the main effect was statistically significant. *P*-values for multiple comparisons were adjusted using Bonferroni's correction. All of the analyses were conducted using STATA data analysis and statistical software (StataCorp LP, College Station, TX). Fisher's exact probability test was used for the retrieval experiments, as previously described (Liang et al., 2014).

## Results

**Figure 2:**
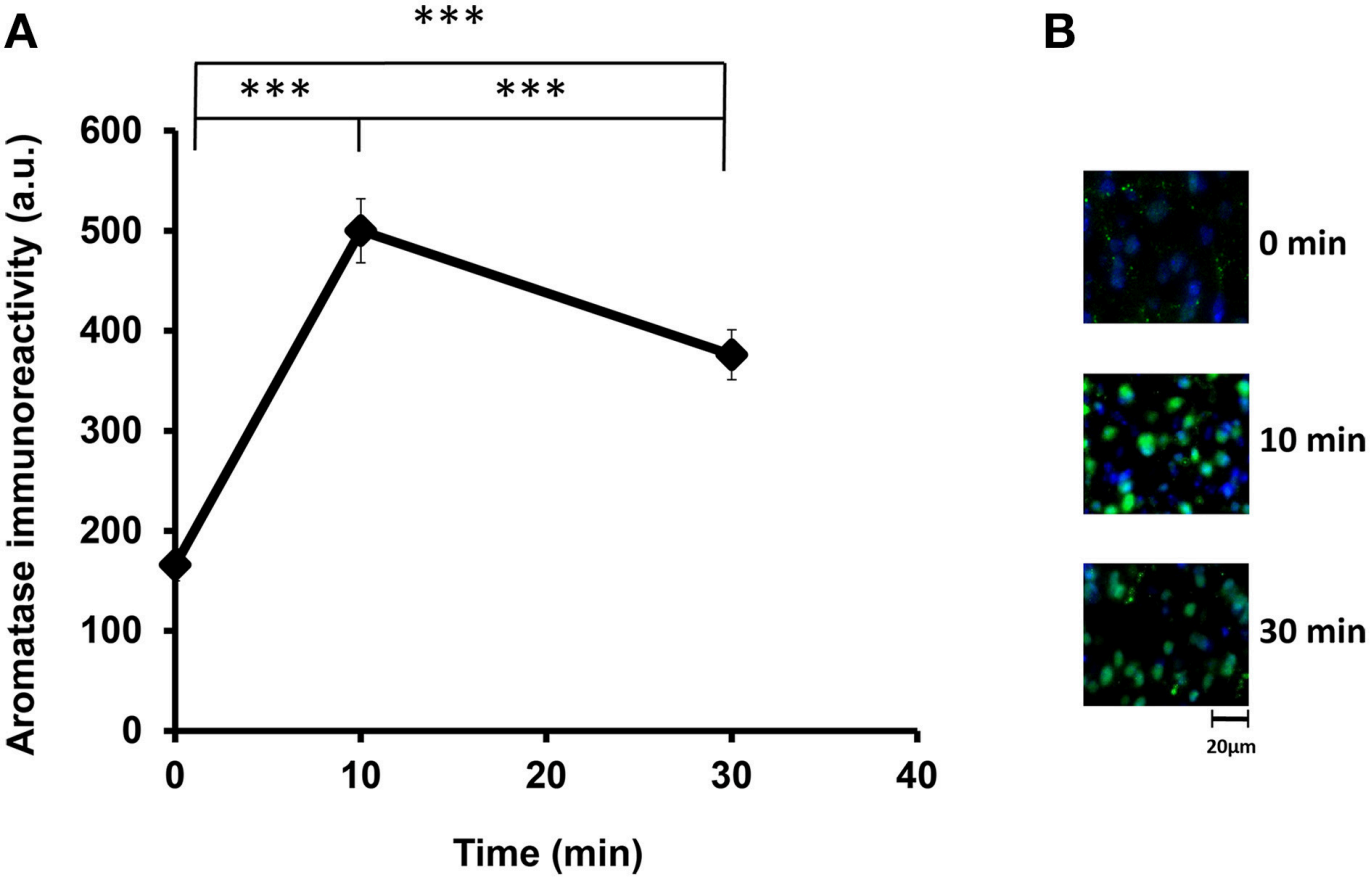
**Time course of the aromatase immunofluorescence intensity after social stimulation**. **(A)** Immunofluorescence was measured in the mPOA regions of sires at 0, 10, and 30 min after isolation together with their pairmate dams in new cages. Three mice were used for each time point. The intensity was measured in 182–421 cells. One-way ANOVA followed by Bonferroni's *post-hoc* test: *F*_(2, 849)_ = 219.82, ^***^P < 0.001. **(B)** Representative images at 0, 10, and 30 min, respectively.

We examined the aromatase immunoreactivity levels in the mPOA region of the sire because it is known that aromatase is abundant in this area, and it is modified rapidly after social stimulation in the mPOA of mice (Veney and Rissman, 2000; Trainor et al., 2003). Presented in Figure 2 are representative images of aromatase immunoreactivity, showing that the intensity increased from 160 activity units (a.u.) at the start of isolation (the base level before separation) to 490 a.u. at 10 min and then declined to 380 a.u. at 30 min [One-way ANOVA followed by a Bonferroni *post-hoc* test, *F*_(2, 849)_ = 219.82, *P* < 0.001]. These results show that the aromatase immunoreactivity can be modified by social stimulation such as father–mother interaction under the parent–pup separation condition. Overall, the changes occurred within a short period, which agreed with previous studies (Konkle and Balthazart, 2011; Cornil et al., 2013). In the following experiments, we fixed the sire brains after separation from the pups for 30 min.

### Immunostaining in various brain regions

**Figure 3:**
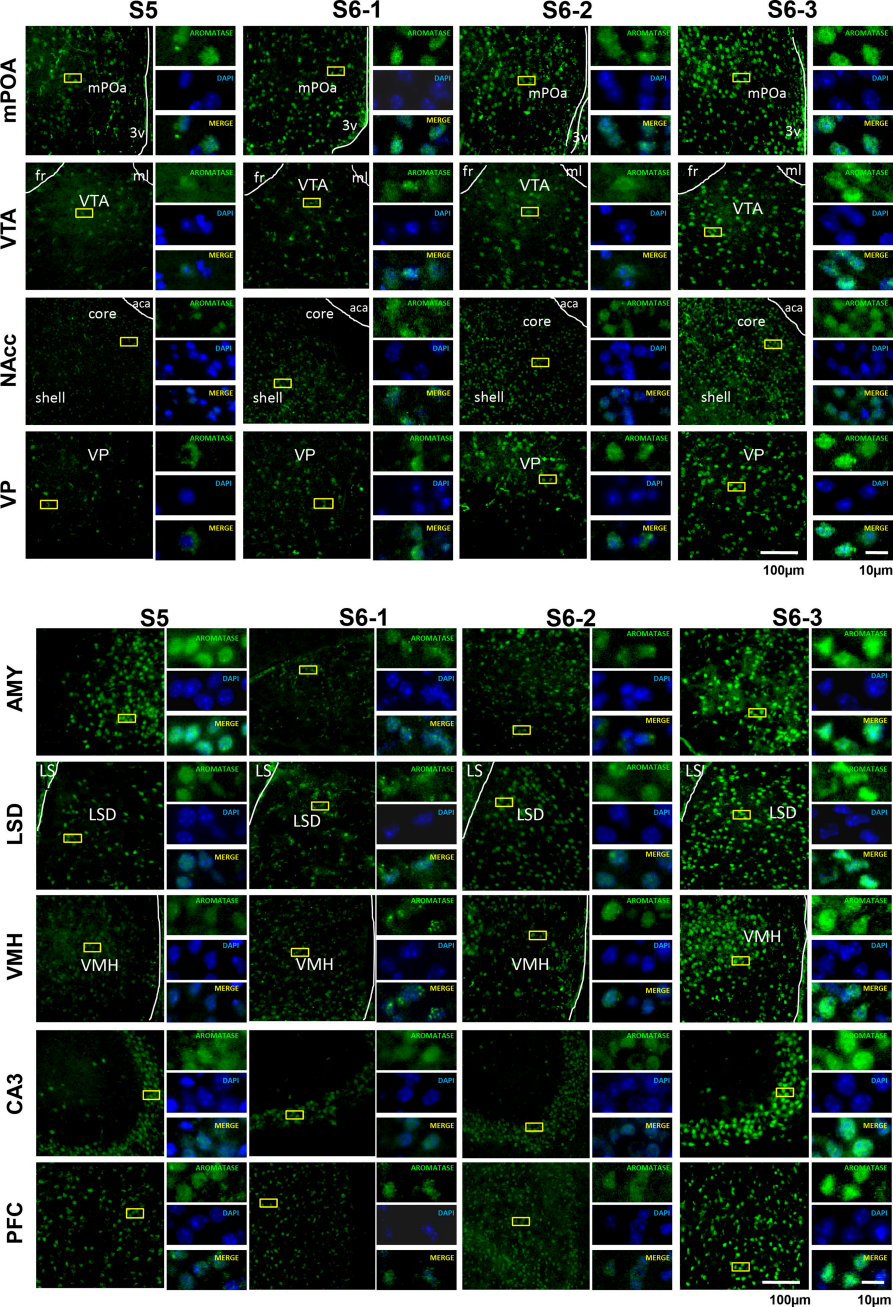
**Photomicrographs of coronal sections showing aromatase immunoreactivity in nine areas of the brains of sires under various housing conditions**. Larger images with lower magnification (left pane) and three smaller images with higher magnifications (right panels) are shown for each brain area on the left. The four housing conditions are indicated at the top. Green (GFP) and blue (DAPI) represent aromatase immunoreactivity and nuclei, respectively. The dashed line indicates the boundary of the third ventricle (3v), fasciculus retroflexus (fr), medial lemniscus (ml), and anterior commissure, anterior part (aca). Scale bars, 100 and 10 μm, respectively.

**Figure 4:**
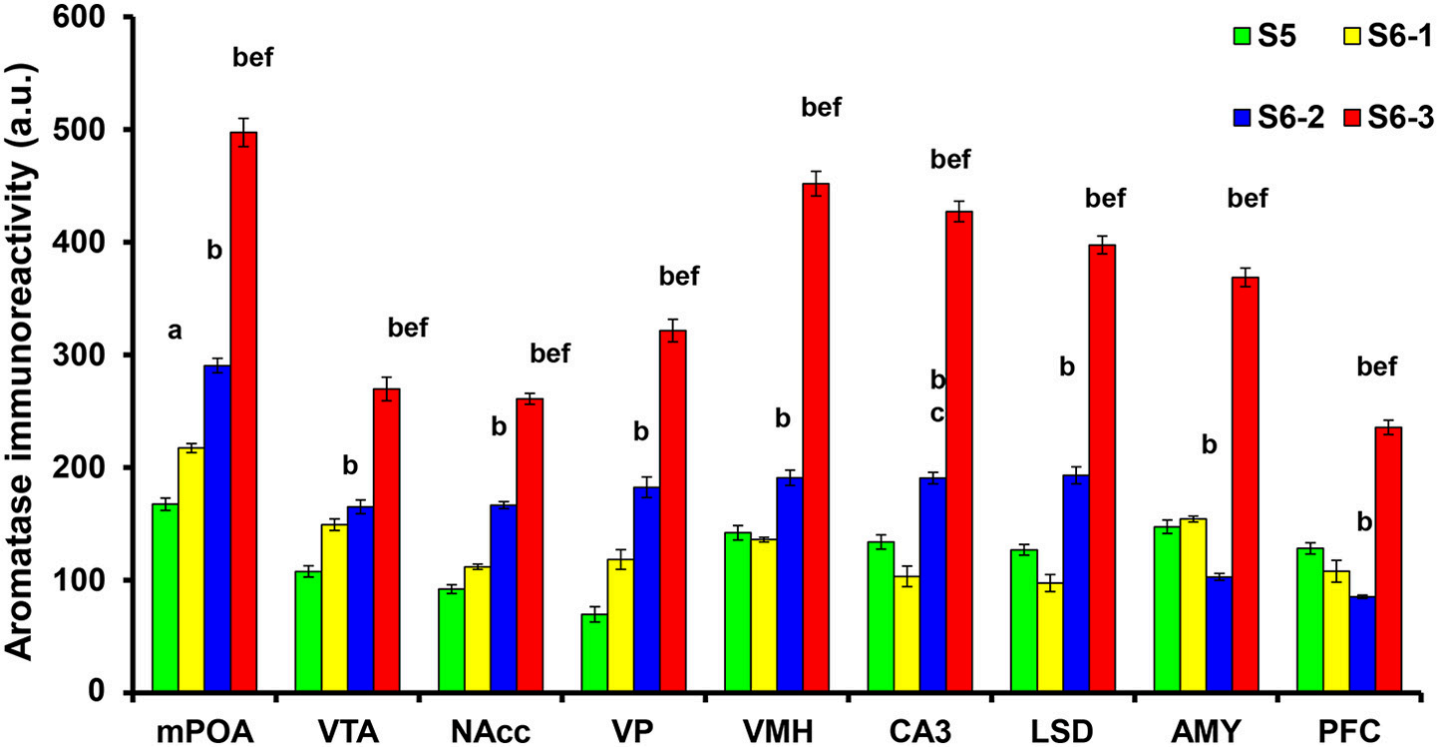
**Statistical analysis of the semiquantitative determination of aromatase expression in different brain regions of sires**. The total intensity of the aromatase signal was measured in each aromatase-expressing cell. The intensity was significantly higher in sires from the S6-3 group, compared with those in the S6-2, S6-1, and S5 groups. The intensity was significantly higher in sires from the S6-2 group, compared with sires from the S5 group in the mPOA, VTA, NAcc, VP, VMH, CA3, and LSD, but lower in the AMY and PFC regions. One-way ANOVA results: *P* < 0.001 in all areas among four conditions (*n* = 642–1430, see text); ^a^*P* < 0.05 vs. S5, ^b^*P* < 0.001 vs. S5, ^c^*P* < 0.05 vs. S6-1, ^d^*P* < 0.01 vs. S6-1,^e^*P* < 0.001 vs. S6-1,^f^*P* < 0.001 vs. S6-2.

We examined the changes in aromatization after 30 min of social stimulation in nine areas of the brain. Figure 3 depicts the representative examples of aromatase staining in the regions of the brains of sires under the three different isolation or control conditions (S5, S6-1, S6-2, and S6-3 in Figure 1). As shown in Figure 4, the most consistent and significant results were that the aromatase immunoreactivity intensity levels were highest in all of the brain areas tested in sires separated in new cages with a pairmate dam (S6-3) compared with the other conditions (S5, S6-1, and S6-2) (One-way ANOVA: mPOA region [*F*_(3, 1139)_ = 233.86, *P* < 0.0001], VTA [*F*_(3, 579)_ = 58.04, *P* < 0.0001], NAcc [*F*_(3, 1430)_ = 309.17, *P* < 0.0001], VP [*F*_(3, 642)_ = 117.43, *P* < 0.0001], VHM [*F*_(3, 1182)_ = 349.40, *P* < 0.0001], CA3 [*F*_(3, 1077)_ = 324.98, *P* < 0.0001], LSD [*F*_(3, 884)_ = 315.65, *P* < 0.0001], AMY [*F*_(3, 1376)_ = 396.90, *P* < 0.0001], and PFC [*F*_(3, 718)_ = 173.04, *P* < 0.0001]).

In seven brain areas out of nine tested, compared with the immunoreactivity in sires in family housing (S5), the immunoreactivity also increased significantly in sires in single housing in a new cage (S6-2). This increase was much less than that in sires housed together with mate dams. This non-negligible increase may be due to environmental effects.

**Figure 5:**
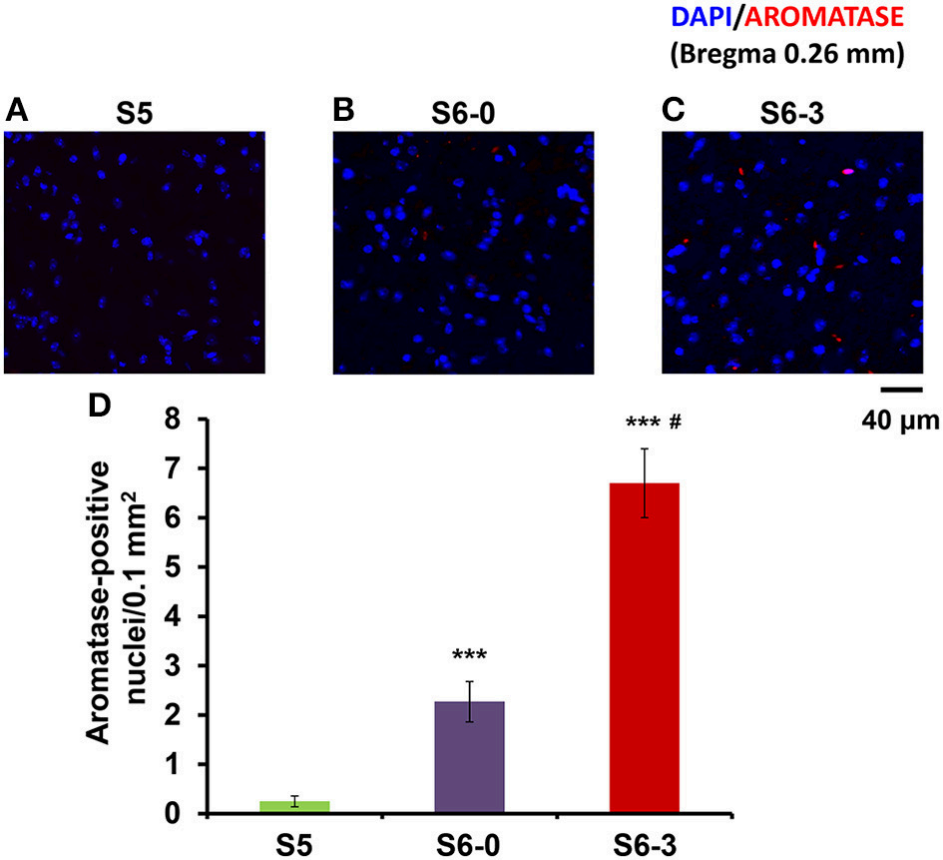
**Photomicrographs of coronal sections showing aromatase immunoreactivity in the mPOA of sires in three different housing conditions**. Each panel shows representative images of a confocal laser-scanning microscope. Cells with aromatase immunoreactivity (red) around the nucleus (blue) in the mPOA of sires in the nursing cage with whole family (S5, **A**), sires with their pairmate dams in old cages (S6-0, **B**) or new cages (S6-3, **C**) after removing pups for 10 min **(B,C)**. **(D)** Statistical analysis of the semiquantitative determination of aromatase expression in different isolation conditions. *N* = 10–16 areas in the mPOA in three mice each. One-way ANOVA followed by Bonferroni's *post-hoc* test: *F*_(2, 33)_ = 47.45, *P* < 0.001; ^***^P < 0.001 from values of sires at S5. ^#^*P* < 0.01 from values of sire at S6-0.

We examined another control in the mPOA of sires to exclude the influence of a new environment by a confocal scanning microscope (Figure 5). The number of nuclei with aromatase immunoreactivity in sires left together with maternal mates in the home cage after pup-removing (S6-0) was significantly larger than that in sires housed with a whole family (S5) and markedly smaller than that in sires isolated together in new cages (S6-3): a One-way ANOVA with Bonferroni's *post-hoc* test, *n* = 3, *F*_(2, 33)_ = 47.45, *P* < 0.0001, ^***^^, #^*P* < 0.001 from values of S5 and S6-0, respectively. The results would underline the hypothesis that maternal mates awakes retrieval in sires and that aromatase is actively suppressed by pups (S5) or alternatively by pups cues (S6-0).

In addition, we compared the aromatase immunoreactivity intensity levels in sires isolated with mate dams in a new cage to that in virgin males and non-retrievers, although the experimental conditions were not exactly the same. The intensity was significantly lower in virgin males (S0) than it was in sires separated with a mate dam (S6-3, Table 1). The intensity was also lower in non-retrievers (S4-1) than it was in sires with mates, except for the AMY, CA5, and LSD regions.

**Table 1:** **Aromatase immunofluorescence intensity in virgin males, non-retrievers, and sires**.

**Area**	**Percentage of the intensity**		
	**Virgin males (S0)**	**Non-retrievers (S4-1)**	**Sires isolated with a mate (S6-3)**
mPOA	42.5 ± 1.6	63.0 ± 1.7	100 ± 2.4^a^^,^ ^b^
VTA	15.0 ± 1.5	33.9 ± 3.1	100 ± 4.7^a^^,^ ^b^
NAcc	37.8 ± 0.8	51.2 ± 2.4	100 ± 1.6^a^^,^ ^b^
VP	13.4 ± 0.5	38.6 ± 3.0	100 ± 2.5^a^^,^ ^b^
VMH	37.0 ± 1.8	17.3 ± 1.9	100 ± 0.6^a^^,^ ^b^
AMY	69.5 ± 1.6	86.5 ± 1.4	100 ± 3.1^a^
CA3	26.0 ± 4.6	83.5 ± 2.3	100 ± 4.7^a^
LSD	40.9 ± 2.5	84.3 ± 2.6	100 ± 3.9^a^
PFC	40.9 ± 3.2	23.6 ± 2.4	100 ± 0.4^a^^,^ ^b^

*The percentage intensity of fluorescence in nine regions of the brains of three different types of males is based on photomicrographs of coronal sections: Virgin males (S0, no separation), non-retrievers after 30 min of retrieval tests in home cages (S4-1), and co-housed sires after 30 min of separation along with mate dams in new cages (S6-3). Values are shown those of sires cohoused with a mate dam as 100%. N = 350 – 1430*.

*A two-tailed Student's t-test*:

a *P < 0.01 vs. S0*;

b *P < 0.05 vs. S4-1*.

### Inhibition of immunoreactivity by sensory deprivation

An independent contrast test of the results given above suggested that the increase in the immunoreactivity intensity was suppressed significantly when sires were pretreated with sensory input deprivation (Table 2) (i.e., by means of preventing auditory signals by filling the ear airway with soft wax, destroying the nasal epithelium with ZnSO_4_, and/or both deprivations, as previously described (Liu et al., 2013). Auditory and olfactory sensory shutdown decreased the intensity of immunoreactivity; One-way ANOVA: mPOA region [*F*_(3, 657)_ = 191.66, *P* < 0.0001], VTA [*F*_(3, 293)_ = 18.93, *P* < 0.0001], NAcc [*F*_(3, 623)_ = 10.72, *P* < 0.0001], VP [*F*_(3, 332)_ = 44.27, *P* < 0.0001], VHM [*F*_(3, 941)_ = 489.86, *P* < 0.0001], CA3 [*F*_(3, 512)_ = 78.50, *P* < 0.0001], LSD [*F*_(3, 521)_ = 262.65, *P* < 0.0001], AMY [*F*_(3, 799)_ = 164.95, *P* < 0.0001], and PFC [*F*_(3, 451)_ = 53.58, *P* < 0.0001]. The inhibition of the immunoreactivity intensity with simultaneous deprivation was additive in the NAcc, VHM, LSD, and PFC regions (the results of the statistical analyses are shown in the figure legends).

**Table 2:** **Aromatase immunofluorescence intensity inhibition by sensory input deprivation in sires**.

**Area**	**Percentage of the intensity compared with that in untreated sires**		
	**Wax**	**ZnSO_4_**	**Wax + ZnSO_4_**
mPOA	26.3 ± 0.4	23.1 ± 1.1	10.6 ± 0.6
VTA	47.5 ± 3.8	45.6 ± 4.1	23.1 ± 2.2
NAcc	58.1 ± 8.8	41.3 ± 4.1^a^	28.8 ± 1.2^b^^,^ ^d^
VP	41.2 ± 2.5	35.6 ± 0.9	21.9 ± 2.6
VMH	30.0 ± 0.8	28.1 ± 1.0	14.4 ± 0.6^b^^,^ ^d^
AMY	39.4 ± 1.6	42.5 ± 1.4	34.4 ± 1.2
CA3	43.8 ± 2.6	40.0 ± 1.4	32.5 ± 2.7
LSD	38.8 ± 2.6	39.4 ± 1.1	11.9 ± 0.5^b^^,^ ^d^
PFC	53.1 ± 1.9	55.0 ± 1.9	33.8 ± 5.2^c^

*The intensity of fluorescence in nine regions of the brains of sires based on photomicrographs of coronal sections after 30 min of separation along with mate dams in new cages (S6-3). The sires were pretreated by filling the ear with soft wax (Wax), pouring ZnSO_4_ in the nose (ZnSO_4_), or both (Wax + ZnSO_4_; Liu et al., 2013). The differences were significant according to a One-way ANOVA followed by Bonferroni's post-hoc test: mPOA region [F_(3, 657)_ = 191.66, P < 0.0001], VTA [F_(3, 293)_ = 18.93, P < 0.0001], NAcc [F_(3, 623)_ = 10.72, P < 0.0001], VP [F_(3, 332)_ = 44.27, P < 0.0001], VHM [F_(3, 941)_ = 489.86, P < 0.0001], CA3 [F_(3, 512)_ = 78.50, P < 0.0001], LSD [F_(3, 521)_ = 262.65, P < 0.0001], AMY [F_(3, 799)_ = 164.95, P < 0.0001], and PFC [F_(3, 451)_ = 53.58, P < 0.0001]*.

a *P < 0.05 vs. wax*.

b *P < 0.001 vs. wax*.

c *P < 0.05 vs. ZnSO_4_*.

d *P < 0.001 vs. ZnSO_4_, respectively*.

### Effects of an aromatase inhibitor on aromatase expression

**Figure 6:**
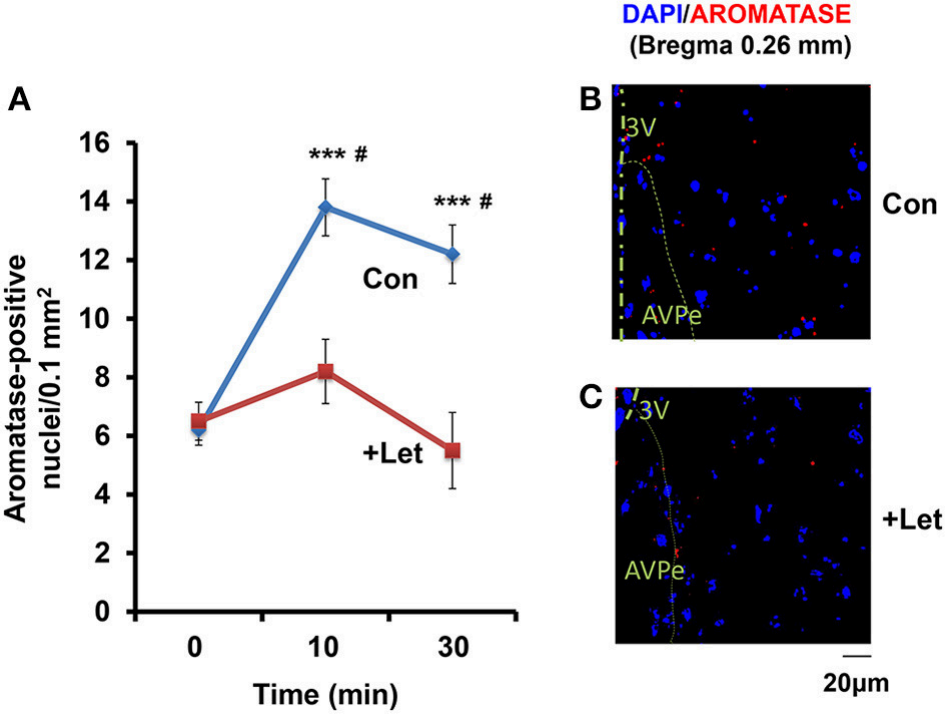
**Time course of the aromatase immunofluorescence-positive cell number in sires treated with letrozole or not**. **(A)** By means of a confocal laser-scanning microscope, the number of cells with aromatase immunoreactivity (red) around the nucleus (blue) was counted in the mPOA regions of sires at 0, 10, and 30 min after isolation together with their pairmate dams in new cages. Five to seven mice were used for each time point. One-way ANOVA followed by Bonferroni's *post-hoc* test: *F*_(2, 12)_ = 20.76, *P* < 0.0001; *F*_(2, 18)_ = 21.85, *P* = 0.1860. ^***^P < 0.001 from values at 0 min. ^#^*P* < 0.001 from values in the presence of letrozole (+Let), two-tailed Student's *t*-test. **(B,C)** Representative images of the mPOA in sires treated with (Let) or without (con) of letrozole, respectively.

Using a fluorescence microscope, we determined whether the aromatase-immunoreactive cell numbers increased. While, the number of reactive cells was highest in all brain regions when tested in the co-housing separation conditions (S6-3), differences in the increase in cell numbers were not as significant among the three separation conditions (S6-1, S6-2, or S6-3; data not shown). Therefore, we used the confocal microscope to detect cells with or without aromatase immunoreactivity, as shown in the representative image (Figures 6B,C). The cell numbers with aromatase-immunoreactivity increased at 10 min and then declined at 30 min (Figure 6A), in a similar fashion when measured as immunofluorescence intensity (Figure 2): A One-way ANOVA with Bonferroni's *post-hoc* test, *n* = 5, *F*_(2, 12)_ = 20.76, *P* < 0.001. Then, we tested the effects of an aromatase inhibitor, letrozole (1 mg/kg of body weight; Geisler, 2011) on the cell number. In the mPOA of sires at 60 min after the intraperitoneal injection of letrozole, the percentage of cell numbers with aromatase did not significantly increase at 10 and 30 min of co-housing with pairmates, respectively: as determined by a One-way ANOVA with Bonferroni's *post-hoc* test, *n* = 7, *F*_(2, 18)_ = 1.85, *P* = 0.163. The percentage cell number at 10 and 30 min in control sires was significantly inhibited, compared to that of sires treated with letrozole, as determined by a two-tailed Student *t*-test, ^#^*P* < 0.01).

### Effects of an aromatase inhibitor on retrieval

Finally, we examined the effects of the aromatase inhibitor on the acquisition of paternity following the parturition of the dams. As shown previously (Liang et al., 2014), when a sire becomes a father, the retrieval behavior increases during the following days spent with pups as a family. As shown previously (Liu et al., 2013), the parental behavior is quickly lost when sires are separated alone in new environment for a few minutes, suggesting that the sire's paternity is reversible, in contrast to the irreversibility of maternity acquisition in dams. Thus, we hypothesized that induction aromatase underlies paternal behavior and such induction is not necessary in maternal behavior.

**Figure 7:**
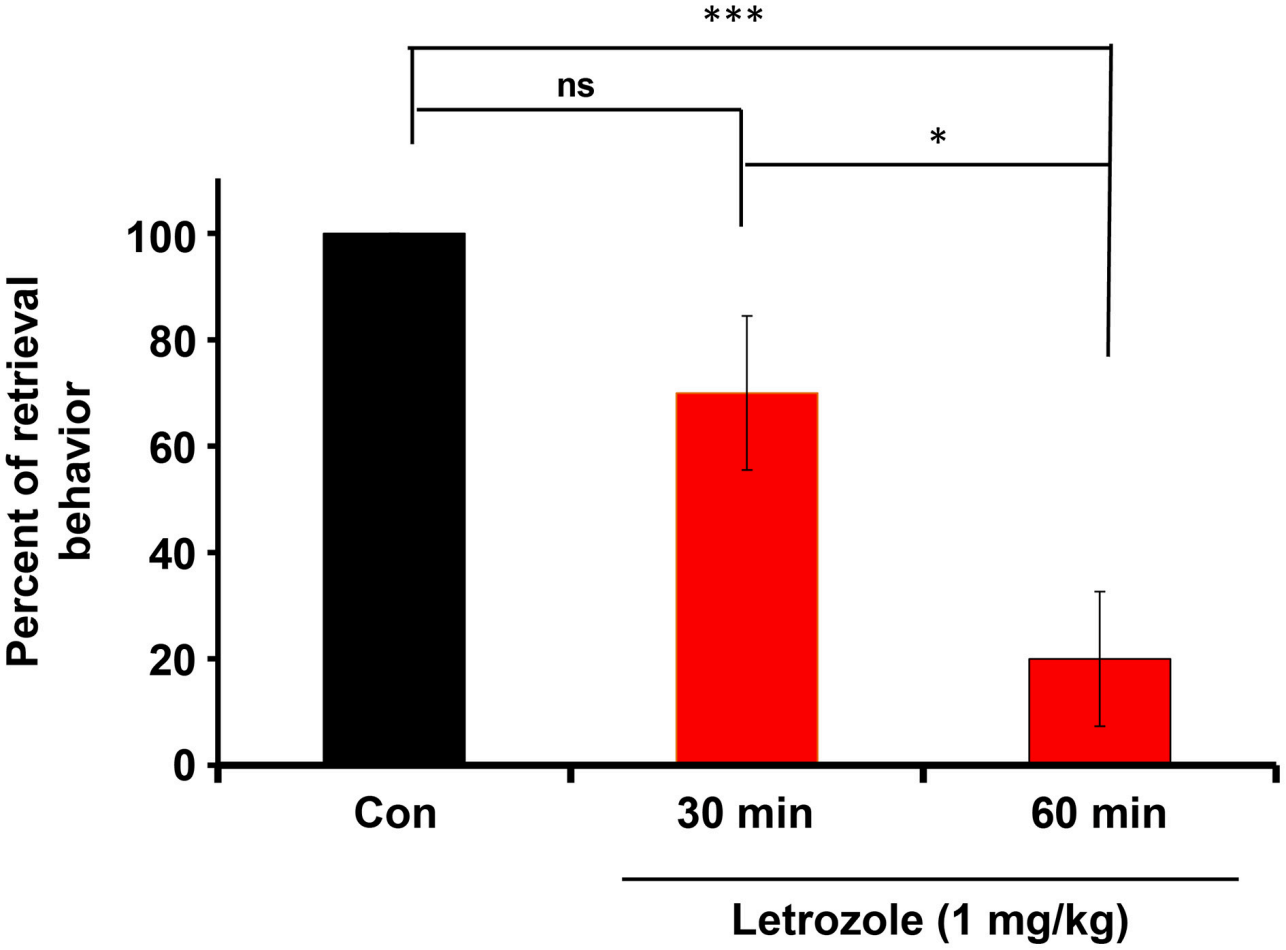
**Inhibition of the co-housing-induced paternal retrieval behavior by an aromatase inhibitor**. The percentage of male parental behaviors was measured based on the retrieval responses after co-habitation with the mother for a 10-min separation period. The couples were housed with their pups for 4 days in their nurturing cages and then removed together from their home cage. Experimental sires were then injected intraperitoneally with letrozole (1 mg/kg of body weight). After 30 or 60 min, the sires were removed along with mates to allow habituation in new cages for 10 min. The sires were returned to the original home with his pups and the retrieval behavior was monitored. *N* = 10 family units in each group. One-way ANOVA followed by Bonferroni's *post-hoc* test detected significant differences between time points: *F*_(2, 27)_ = 11.92, *P* < 0.001, *n* = 10 in each group. ^*^P < 0.05, ^***^P < 0.001.

The couples were housed with their pups for 4 days in their nurturing cages. Experimental sires were then injected intraperitoneally with letrozole (1 mg/kg of body weight). After 30 or 60 min, the sires were isolated together with their pairmates to allow habituation in new cages for 10 min. The sires were returned to the original home with his pups and the retrieval behavior was monitored. The effect of letrozole on retrieval was evident at 60 min (Figure 7): a One-way ANOVA followed by Bonferroni's *post-hoc* test detected significant differences between time points: *F*_(2, 27)_ = 11.92, *P* < 0.001, *n* = 10 in each group; ^*^P < 0.05, ^***^P < 0.001.

**Figure 8:**
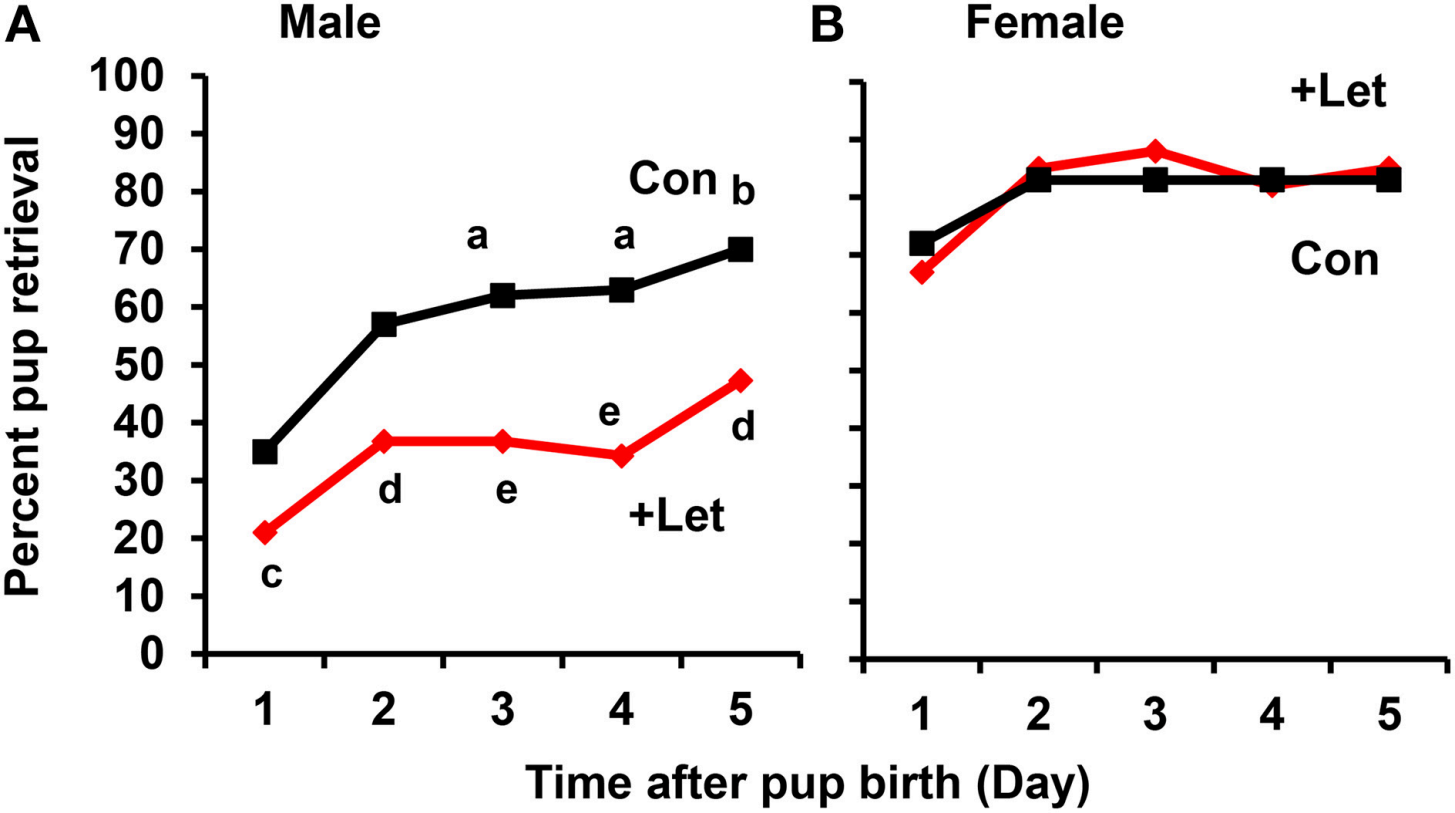
**Effect of letrozole on parental retrieval behavior development**. The capacity for pup retrieval by first-time sires **(A)** and primiparous dams **(B)** was measured daily from day 1 to day 5 after parturition. The sires (*n* = 31) were administered peritoneally each day with letrozole (1 mg/kg of body weight, *n* = 57–67) or PBS (*n* = 30–31). The dams were also treated in a similar manner with letrozole (*n* = 26–31) or PBS (*n* = 5–9). After 1 h, the retrieval behavior per 10 min was measured, which was repeated for 5 days. The number of pups retrieved was counted and expressed as a percentage. Fisher's exact test, two-tailed:^a^*P* < 0.05, ^b^*P* < 0.02 vs. day 1; ^c^*P* < 0.05,^d^*P* < 0.01, ^e^*P* < 0.001 between treated and untreated sires.

To assess acquisition of paternal behavior, the sires and dams were injected once with letrozole (1 mg/kg of body weight) on the first day after the dam's parturition and on the 4 subsequent days. At 50 min after letrozole injection, both parents were separated from their pups together in new cages for 10 min, and the retrieval behaviors of both parents were then examined for 10 min using a previously described protocol (Liu et al., 2013). In the untreated sires, the retrieval behavior increased significantly on day 3 (*P* < 0.02) and on day 5 (*P* < 0.05) after the first day, as revealed by a two-tailed Fischer's exact test, *n* = 57–67 at each time point. In contrast, in the letrozole-treated sires, the retrieval behavior was suppressed significantly each day (*P* < 0.05 on day 1 and 5, *P* < 0.01 on day 2, *P* < 0.001 on days 2–5), compared to that of untreated sires, *n* = 31–67 at each time point; Figure 8A). These results clearly show that retrieval changes as a function of day in untreated sires and aromatase inhibition impairs retrieval at all times in treated sires. In sharp contrast, maternal retrieval was not impaired by the treatment (Figure 8B).

## Discussion

In this study, we determined the distribution of aromatase-containing cells in nine regions of the brains of retriever sires in the ICR mouse. The aromatase immunoreactivity intensity was enhanced in these brain areas in the sires within minutes by social stimulation (i.e., separation from pups along with their paired dams). There observed a large suppression of aromatase when all (mom and pups) are present (S5) compared to being alone in the home cage (S6-1) and that aromatase is desinhibited by a novel cage (removal of pup and dam cues, S6-2) and further activated by the presence of the dam (S6-3). Alternatively, aromatase activity was highest when the sires were in the presence of the dam, and, more important, when the pups were absent. It is therefore plausible that the dam significantly stimulates aromatase in the male brain, or that the presence of the pups has an inhibitory effect on this increase, though the later possibility was not intensively examined. These immunoreactivity increases in sires were suppressed by sensory input reductions caused by physical auditory and chemical olfactory interventions, which effectively prevented communication with pairmate dams (Liu et al., 2013). The aromatase intensity in non-retrievers was higher in several regions, compared with that in virgin males (S0), which appeared to be the basal level (Table 2). Treatment of sires with the aromatase inhibitor letrozole (Geisler, 2011) effectively decreased the aromatase immunoreactivity-positive cell number, suggesting that aromatase immunoreactivity might be correlated with activity, although this is only a demonstration of correlation. Letrozole suppressed the development of the paternal retrieval behavior during the first 5 days after parturition by the dams, whereas a single dose of the inhibitor had no effects on the retrieval behavior of dams. This agrees well with a previous study, which showed that aromatase inhibition by letrozole reduced paternal behavior in hamsters (Timonin and Wynne-Edwards, 2008).

Several lines of evidence indicate that rapid estrogen-mediated signaling (ranging from seconds to minutes) has potent effects on molecular and cellular events; thereby, resulting in the “fine-tuning” of the neuronal circuitry (Balthazart et al., 2001, 2005). Among vertebrate species, one of the estrogens, particularly 17β-estradiol (E2) acts on the brain via both genomic and non-genomic mechanisms to influence neuronal physiology and behavior (Cornil et al., 2006). Non-genomic signaling is typically initiated by membrane-associated estrogen receptors that modulate intracellular signaling cascades. Thus, brain-synthesized estrogens, which are mediated by the enzyme aromatase, may be the source of rapid estrogen effects within the brain (Balthazart and Ball, 1998, 2006; Charlier et al., 2010, 2013; Azcoitia et al., 2011). It is well known that aromatase is found in two locations (Jakab et al., 1993; Naftolin, 1994), the hypothalamus and limbic systems, and it regulates a variety of social behaviors, including courtship, aggression, and sexual behavior. For the first time, we propose that aromatase also mediates the tuning of paternal behavior.

**Figure 9:**
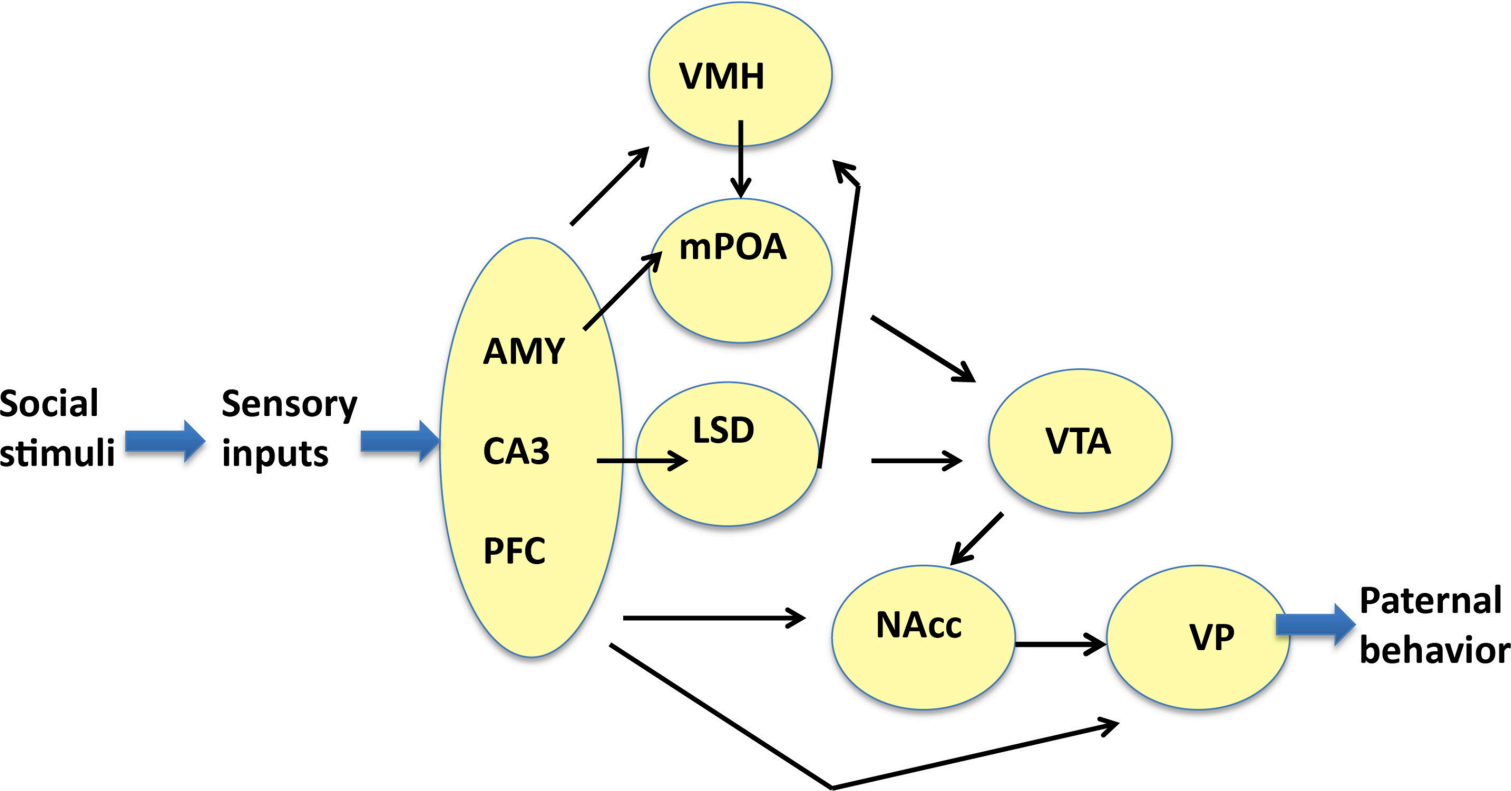
**Neural model showing the circuits that may regulate the paternal retrieval behavior**. This figure is based on Sheehan et al. (2004), Zhong et al. (2014), Akther et al. (2014), Numan et al. (2005), Rilling and Young (2014), Numan (2015), and Bridges (2015). The figure shows how aromatase-expressing neurons relay signals related to social stimuli, which convert the sensory information received by sires to elicit the retrieval behavior. The sensory inputs are processed in the prefrontal cortex (PFC), CA3 region of the hippocampus (CA3), and medial amygdala (AMY), which are grouped. The output signals from the three regions are transmitted to the ventromedial hypothalamus (VMH), medial preoptic area (mPOA), and lateral septum dorsal (LSD), followed by the ventral tegmental area (VTA), and nucleus accumbens (NAcc). The final region is the ventral pallidum (VP), which induces motion. Information related to neurotransmitters and excitatory or inhibitory regulators is omitted for simplicity.

Enhancements of the intensity of aromatase immunoreactivity by communicative interactions have been observed widely, including four brain regions, i.e., the mPOA, VTA, NAcc, and VP, which are the primary parental brain regions related to pup care (Sheehan et al., 2004; Olazábal et al., 2013; Rilling and Young, 2014; Bridges, 2015; Numan, 2015), and five other regions, i.e., the AMY, LSD, VMH CA3, and PFC (Figure 9). However, among the four important primary areas known to be involved in nursing behavior (Bridges, 2015; Numan, 2015), there were no increases in the VMH, AMY, and CA3 regions. The detection of aromatization in the brains of non-monogamous sires in the current study provides novel insights into brain feminization because these regions may contain the brain network related to paternity (paternal care behavior).

Our detection of rapid effects on the immunoreactivity of aromatase and of the inhibitor on retrieval resemble the typical behavior observed in marmoset fathers, which is associated with rapid estrogen synthesis and the rapid onset of paternal parental care in response to olfactory cues from dependent children (Ziegler et al., 2011), where the time required for paternal behavior initiation is < 10 min. This rapid time window for typical behaviors in males with a latency of 10–15 min has been reported frequently, and it is controlled rapidly by brain-derived estrogens in other species (Charlier et al., 2013). Thus, it is reasonable to suggest that the paternal retrieval behavior is due to the synthesis of estrogen or at least modulated by estrogens. This modulation by estrogens is known to allow a rapid non-genomic control of the activity of aromatase in the brain (Cornil et al., 2006). However, in quail brains, the change in the enzyme activity is much higher than the change estimated based on the immunoreactive cell number and aromatase mRNA; thereby, suggesting that some other form of enzyme change may also have an important role. The activity of the enzyme is regulated by a Ca^2+^-dependent phosphorylation–dephosphorylation step (Balthazart et al., 2001, 2005). Aromatase is activated by dephosphorylation and disinhibition with calmodulin in the vertebrate brain (Charlier et al., 2013). It has been shown that the intensity of immunoreactivity reflects the enzyme activity well (Suzuki et al., 1994), so the increase in the immunointensity after social interaction between dams and sires probably reflects this increased enzyme activity; however, further careful examinations are required to support this hypothesis. One possibility to be investigated in such studies is the double staining of aromatase in activated cells (e.g., using Fos), which could provide valuable information on the type of activated areas as a function of cues in dam, pups, or both. Another possible study is the detection of aromatase mRNA levels in this time frame. Currently, these studies are ongoing.

Our results suggest that aromatase-rich neurons in the brain produce estradiol in the cytoplasm. Intraneuronal estrogen receptors may be activated by this product and mediate physiological roles, including paternal behavior. The functional significance of intracellularly produced estrogens acting in neurons remains an open question. However, it has been proposed that the p21-activated kinase, extracellular signal-regulated kinase, phosphatidylinositol 3-kinase, or CRE-binding proteins are phosphorylated as non-genomic mechanisms (Srivastava et al., 2010; Fester and Rune, 2015; Zhu et al., 2015), resulting in modulation of synaptic signaling or structure and brain development (Peruffo et al., 2011). These mechanisms may well explain our finding that the aromatization-contributed sensitization of the pup retrieval behavior in sires occurred rapidly during the first 3 days after the parturition of dams, because the aromatase inhibitor, letrozole, significantly suppressed the retrieval behavior each day and slowed the acquisition of this ability (paternity).

We found higher aromatase levels in nine regions in the brains of sires that experienced communicative interactions (S6-3) compared with the sires that lacked communicative interactions; thereby, suggesting a possible role for a paternity network in addition to the specific function of each area (Figure 9). In the mPOA, estradiol promotes paternal behavior in male California mice (Trainor and Marler, 2002). The mPOA could become more sensitive to estradiol via a change in the receptor number because of a study on a paternal strain of domestic mice found that more cells contained estrogen receptors in the mPOA of reproductively experienced males compared with virgin males (Ehret et al., 1993). In the VTA, estrogens have also been shown to affect catecholaminergic neurons directly via non-genomic mechanisms (Numan and Stolzenberg, 2009; Stolzenberg and Numan, 2011). Recently, it was reported that aromatase-expressing neurons within the male posterodorsal medial AMY regulate the components of aggression, but not other estrogen-dependent male-typical behaviors (Unger et al., 2015).

In conclusion, these data unequivocally demonstrate that rapid estrogen synthesis is associated with the neural activation of the paternal social (nurturing) brain in the sires and with the sensitization of male parental behavior (development of fatherhood) after becoming a father.

### Conflict of interest statement

The authors declare that the research was conducted in the absence of any commercial or financial relationships that could be construed as a potential conflict of interest.
